# Impact of frailty on the outcomes of patients undergoing degenerative spine surgery: a systematic review and meta-analysis

**DOI:** 10.1186/s12877-023-04448-2

**Published:** 2023-11-23

**Authors:** Wonhee Baek, Sun-Young Park, Yoonjoo Kim

**Affiliations:** 1 College of Nursing, Gyeongsang National University, Jinju-si, Gyeongsangnam-do South Korea; 2 College of Nursing, Daegu Catholic University, Daegu-si, South Korea; 3 Department of Nursing, College of Healthcare Sciences, Far East University, Eumseong-gun, Chungcheongbuk-do South Korea

**Keywords:** Frailty, Meta-analysis, Patient-reported outcome measures, Spine surgery, Systematic review

## Abstract

**Background:**

Degenerative spinal diseases are common in older adults with concurrent frailty. Preoperative frailty is a strong predictor of adverse clinical outcomes after surgery. This study aimed to investigate the association between health-related outcomes and frailty in patients undergoing spine surgery for degenerative spine diseases.

**Methods:**

A systematic review and meta-analysis were performed by electronically searching Ovid-MEDLINE, Ovid-Embase, Cochrane Library, and CINAHL for eligible studies until July 16, 2022. We reviewed all studies, excluding spinal tumours, non-surgical procedures, and experimental studies that examined the association between preoperative frailty and related outcomes after spine surgery. A total of 1,075 articles were identified in the initial search and were reviewed by two reviewers, independently. Data were subjected to qualitative and quantitative syntheses by meta-analytic methods.

**Results:**

Thirty-eight articles on 474,651 patients who underwent degenerative spine surgeries were included and 17 papers were quantitatively synthesized. The health-related outcomes were divided into clinical outcomes and patient-reported outcomes; clinical outcomes were further divided into postoperative complications and supportive management procedures. Compared to the non-frail group, the frail group was significantly associated with a greater risk of high mortality, major complications, acute renal failure, myocardial infarction, non-home discharge, reintubation, and longer length of hospital stay. Regarding patient-reported outcomes, changes in scores between the preoperative and postoperative Oswestry Disability Index scores were not associated with preoperative frailty.

**Conclusions:**

In degenerative spinal diseases, frailty is a strong predictor of adverse clinical outcomes after spine surgery. The relationship between preoperative frailty and patient-reported outcomes is still inconclusive. Further research is needed to consolidate the evidence from patient-reported outcomes.

**Supplementary Information:**

The online version contains supplementary material available at 10.1186/s12877-023-04448-2.

## Background

As the incidence of degenerative spinal diseases has increased and with advancements in medical technology [1, 2], the number of older adults undergoing spine surgeries has increased [3, 4]. Accordingly, difficulties encountered during spine surgeries have also increased [4, 5]. Because the outcomes of patients undergoing spine surgery are affected by their preoperative characteristics [6–8], it becomes imperative to gain insights into factors that may impact postoperative outcomes in this population, including frailty. Frailty is defined as a multidimensional state of loss of physical, cognitive, social, and psychological functioning [9]. The older the age, the higher the frailty; however, compared to chronological age, frailty status can better predict complications and mortality following spine surgery [10]. Most patients undergoing spine surgeries are prefrail or frail [7, 11], conditions which are often associated with preoperative pain, spinal deformity, and reduced ability to perform activities of daily living. For spine surgery, the incidence of postoperative complications and non-home discharge, length of hospital stay, and mortality rates are higher among patients with preoperative frailty than among those without [7, 12]. Therefore, preoperative risk stratification of frailty is helpful for predicting postoperative deterioration; this in turn can help prevent the worsening of outcomes after a spine surgery [9].

Patients with frailty who have undergone spine surgery do not experience the same level of benefit in terms of clinical outcomes (COs) as those who are not frail [13, 14]. Even then, such patients often opt for spine surgery to alleviate pain and improve function rather than for survival (unlike patients who opt for cancer surgery) [15]. Therefore, providing patients with information on the benefits of patient-reported outcomes (PROs) after spine surgery can help them make informed decisions and receive more patient-centred care. With the increased emphasis on the importance of PROs, research has increasingly focused on how PROs in frail patients have changed following spine surgery [13, 16]. However, there is a lack of understanding of the benefits and expected types of PROs in spine surgery. Therefore, a systematic literature review and meta-analysis of the relationship between preoperative frailty and the postoperative outcomes of surgery for patients with degenerative spinal disease is necessary.

A 2021 systematic review and meta-analysis of 32 studies on preoperative frailty and outcomes of spine surgery revealed that frailty was associated with increased adverse events, mortality, length of hospital stay, readmission, reoperation, non-home discharge, intensive care unit stay, and PROs following a spine surgery [17]. However, this review had the following limitations: studies on simple procedures such as kyphoplasty were included in the review; therefore, the risk of bias regarding non-surgical procedures could not be ruled out. Furthermore, because disease pathogenesis and progression differ between patients with spinal neoplasms and metastases and those with degenerative spine disease, both cohorts must be analysed separately. However, the study mentioned above included both patients with spinal neoplasms and those with degenerative spinal diseases. Moreover, interpretation of the findings of the meta-analysis was limited because the postoperative adverse events were not differentiated in detail, a synthesis of evidence on the patient-reported outcomes was not performed, and the method for the meta-analysis was not described clearly [17–19].

Two parameters help to identify frailty status. These include the frailty phenotype [20] and the frailty index (FI) [21]. Regarding the frailty phenotype, frailty is determined by the following symptoms: unintentional weight loss, self-reported exhaustion, weakness, slow walking speed, and low physical activity [20]. The FI is obtained by dividing the sum of a patient’s deficits by the total sum of frailty-related deficits. It has two types, namely adult spinal deformity (ASD)-FI [13] and cervical deformity (CD)-FI [22]. Recently, modified FI (mFI) has also been used for determining frailty [23]; each clinical institution has developed and used a different frailty tool [24]. Determining the risk stratification of frailty before spine surgery helps determine the prognosis and treatment of patients. Thus, we aimed to explore the following: (1) tools used to measure the frailty of patients prior to surgery for degenerative spine disease, (2) types of frailty-related health-related outcomes following spine surgery, and (3) association between preoperative frailty and health-related outcomes.

## Methods

We followed the recommendations of the Cochrane Handbook to confirm the outcome of frailty [25]. The final protocol was registered in the International Prospective Register of Systematic Reviews (PROSPERO; registration number: CRD42021286341).

### Search strategy

Electronic bibliographic databases, including Ovid-MEDLINE, Ovid-EMBASE, Cochrane Library (Cochrane Database of Systematic Reviews), and CINAHL (Cumulative Index of Nursing and Allied Health), were screened for relevant articles. The search terms were “spine,” “frailty,” “postoperative,” and “outcome” and the Boolean operators OR and were used to combine them. The search was completed on July 16, 2022. The search strategies for each database are presented in Supplementary Material Table 1.

### Eligibility criteria

The inclusion criteria were as follows: (1) articles on patients who underwent spine surgery; (2) articles on studies that compared health-related outcomes (COs and PROs) after spine surgery with respect to preoperative frailty status, (3) articles in English published in peer-reviewed journals; and (4) articles on prospective or retrospective cohort, case-control, and cross-sectional studies. The exclusion criteria were as follows: (1) reviews, case reports, and unpublished manuscripts; (2) articles on studies that included spinal tumours; (3) articles on experimental studies (interventions could confound the relationship between frailty and postoperative health-related outcomes); (4) articles on studies that included non-surgical procedures. No restrictions were placed on the timing of publication.

### Article selection and data extraction

Articles were first downloaded using reference management software (EndNote version 20, Clarivate Analytics, USA). Then, Rayyan was used to screen the downloaded articles and remove any duplicates [26]. Two authors (WB and YK) independently read the titles and abstracts of the remaining articles and selected those that met the eligibility criteria. Thereafter, the full texts of the selected articles were reviewed; any discrepancies in the selection process were resolved after discussion with another author (SP). Using a standardized record extraction form, the two aforementioned reviewers independently extracted the following data from the selected articles: first author’s name, year and country of publication, demographic and clinical characteristics of the study population, population demographics, type of surgery, measurement tool and outcomes, and follow-up duration.

### Risk of bias in individual studies

The Risk of Bias Assessment Tool for Nonrandomized Studies (RoBANS) was used to assess the quality of the included studies [27]. The RoBANS evaluated the risk of bias for the following six domains: participant selection, confounding variables, measurement of exposure, blinding of outcome assessments, incomplete outcome data, and selective outcome reporting. Each domain was assessed as having a “low risk of bias”, “unclear risk of bias,” or “high risk of bias.” The two aforementioned authors independently evaluated the methodological quality of the studies and later combined their findings.

### Synthesis and statistical analysis

All data analyses were performed using R (version 4.0.3, R Foundation for Statistical Computing, Austria). We performed a qualitative synthesis to determine what tools were used to measure frailty in patients undergoing spine surgery and what indicators were used for frailty and health-related outcomes. Thereafter, quantitative synthesis was performed to confirm the direction and magnitude of the association between frailty and health-related outcomes.

We divided the postoperative health-related outcomes into COs and PROs. The meta-analysis was performed if the following conditions were met: (1) there were three or more papers that could be synthesized, (2) the participants could be divided into frail and non-frail groups, (3) COs were synthesized only if the terms used in each paper were identical, and (4) the same participants were extracted from the same database in the same year (the paper that was published first was selected).

The Mantel–Haenszel method was used to estimate the pooled odds ratio (OR) with the 95% confidence interval (CI) for dichotomous variables. The inverse variance method was used to estimate the pooled mean difference (MD) with the 95% CI for continuous variables. A fixed-effect model was used for homogeneous studies, while a random-effects model was used for heterogeneous studies [25]. The *I*^*2*^ value was used to investigate the heterogeneity among the included studies; an *I*^*2*^ value > 50% was considered indicative of substantial heterogeneity [28].

Because tests for publication bias need to be evaluated when there are more than 10 studies in a meta-analysis, statistical tests were not attempted to identify publication bias in our study. Sensitivity analysis was performed while excluding papers that were judged to increase the heterogeneity and cause a bias in the effect size in the meta-analysis [25]. Statistical significance was defined by p-value < 0.05.

## Results

### Study selection

**Fig. 1:**
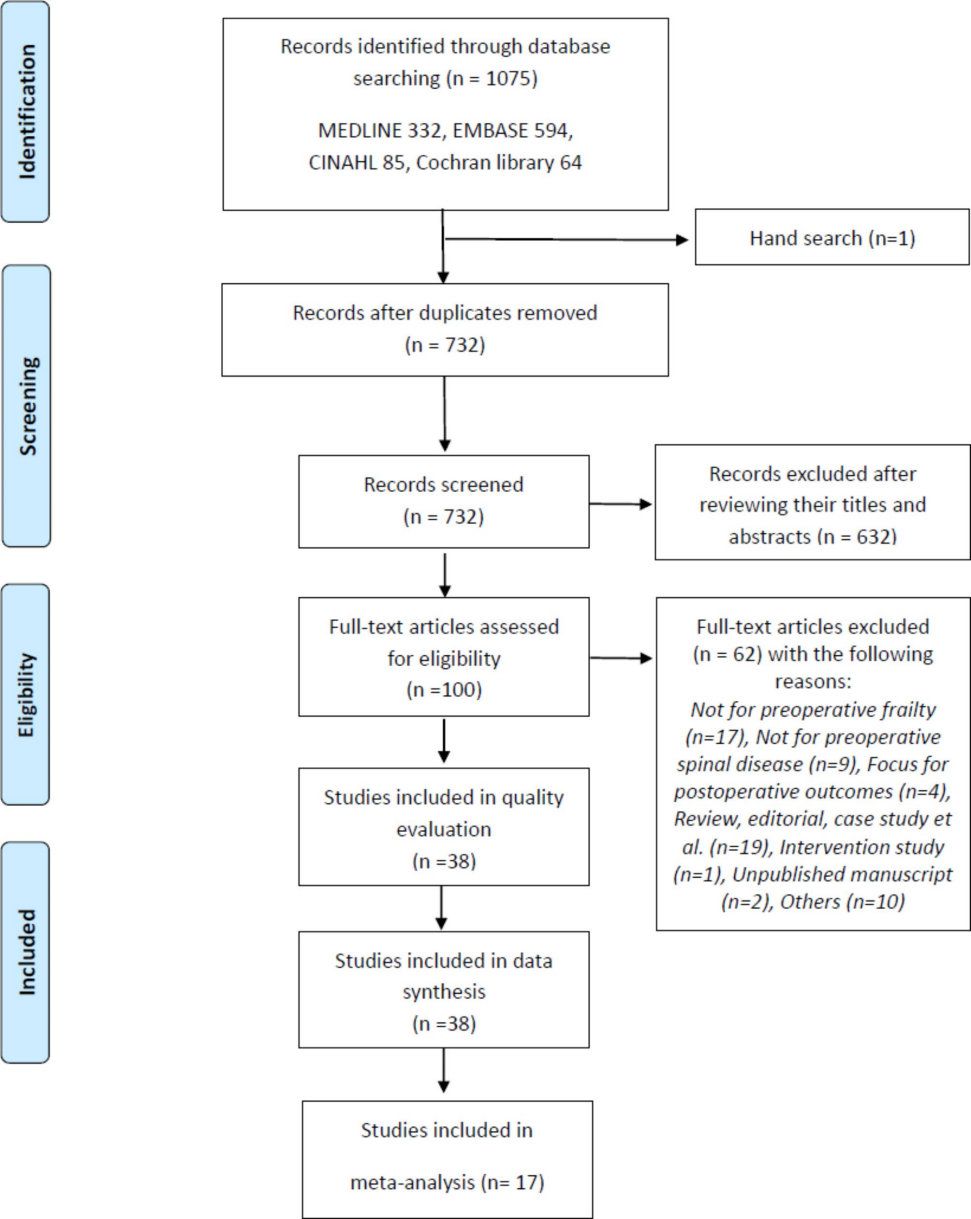
Preferred reporting items for systematic reviews and meta-analyses-based flowchart of the article screening and selection process

The study selection process is shown in Fig. 1. The initial search of the databases yielded 1,075 potentially relevant articles; one additional article was identified from other sources [29]. Among these, 732 articles remained after the removal of duplicates. After screening their titles and abstracts, 632 of these articles were excluded. The full texts of the remaining 100 articles were reviewed, and 62 articles were further excluded. The remaining 38 articles were finally included for quality evaluation and qualitative synthesis [7, 10–14, 16, 22–24, 29–56]. Among these, 17 were subjected to a quantitative synthesis for the meta-analysis [10, 13, 16, 22, 29, 30, 33, 35, 39–42, 47, 49, 52, 55, 56].

### Study characteristics

The characteristics of the included studies are presented in Table 1. The countries of the patients who participated in the study were North America (n = 25) [7, 10–14, 22–24, 29, 31, 32, 37, 40–45, 47–49, 51–53, 56], Korea (n = 5) [30, 33–36], China (n = 2) [16, 50], Europe (n = 2) [38, 46], Japan (n = 2) [54, 55]. One study included patients from Europe, Asia, and North America [39]. Overall, 34 retrospective cohort studies [7, 10, 13, 14, 16, 22–24, 29, 31–38, 40–47, 49–56], 3 prospective cohort studies [11, 30, 39, 48], and 1 mixed retrospective and prospective cohort study [12] were included. The articles were published between 2016 and 2022. Overall, the studies comprised 474,651 patients who underwent spine surgery (mean age: 56.6–78.3 years).

### Risk of bias

Supplementary Material Fig. 1 summarizes the results of the assessments of the risk of bias in the included studies. The overall quality of the included studies was good. However, there were concerns regarding selection bias for six out of 38 studies [23, 29, 36, 45–47]. These studies analysed multi-centre data and had a retrospective design, but did not report the confounding variables. Eleven studies [10, 14, 16, 22, 23, 29, 34, 35, 40, 52, 53] did not report the presence of incomplete outcome data, such as missing data or non-response rates. In more than 80% of the studies, five of the six evaluated domains were assessed as having a low risk of bias (attrition bias was excluded). No studies were excluded based on quality assessment.

### Frailty measurements

The measurement tools for preoperative frailty included the mFI-11 (n = 15) [10, 12, 16, 23, 30, 32, 33, 35, 41, 44, 49, 50, 53–55], mFI-5 (n = 10) [7, 30, 31, 34, 44, 45, 52, 53, 55, 56], ASD-FI (n = 6) [13, 37–39, 42, 47], Hospital Frailty Risk Score (n = 2) [14, 46], Johns Hopkins Adjusted Clinical Groups indicator (n = 2) [24, 51], mCD-FI (n = 2) [29, 43], frailty phenotype (n = 3) [11, 36, 48], CD-FI (n = 1) [22], comprehensive geriatric assessment (n = 1) [30], and mASD-FI (n = 1) [40]. In these studies, the patients were divided into non-frail, prefrail, frail, or severely frail groups or into the low frailty, medium frailty, and high frailty groups, according to their criteria.

### Health-related outcomes after spine surgery

**Fig. 2:**
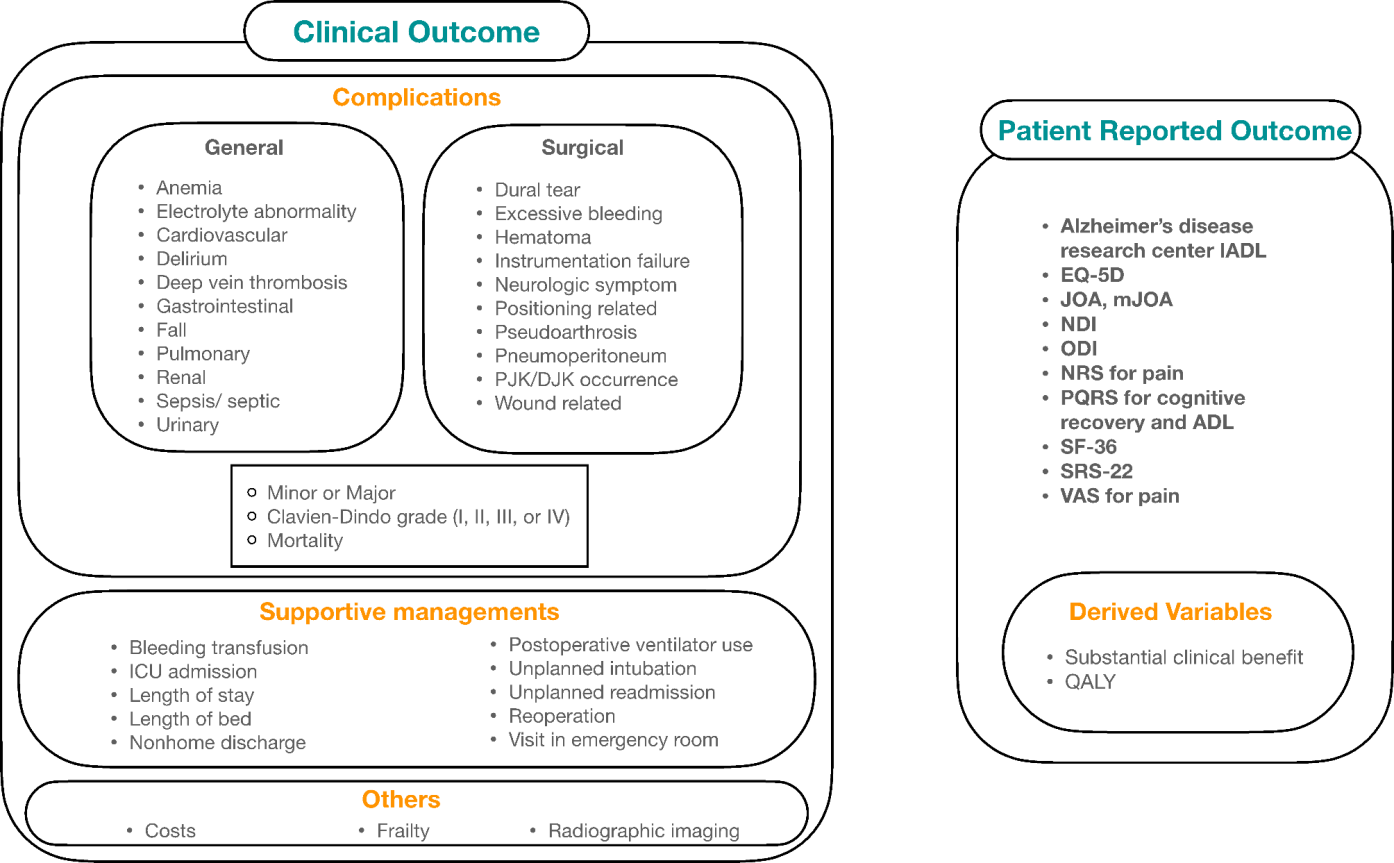
Health-related outcomes in terms of preoperative frailty status. IADL, instrumental activities of daily living; EQ-5D, EuroQol-5D; JOA, Japanese orthopedic association scale; mJOA, modified Japanese orthopedic association scale; NDI, neck disability index; ODI, Owestry disability index; NRS, numerical rating scale; PQRS, postoperative quality of recovery scale; ADL, activity of daily living; SF-36, 36-item short-form survey; SRS-22, Scoliosis Research Society 22-question; VAS, visual analog scale; QALY, quality-adjusted life years; ICU, intensive care unit

In the included studies, postoperative health-related outcomes were classified into COs and PROs (Table 1; Fig. 2, and Supplementary Material Table 2).

**Table 1:** Characteristics of the included studies

Author (year)	Country	Design (database, acquired date)	Surgery	Sample size; age (years)	Frailty tool	Cut-off value (frailty grouping)	Postoperative outcomes		Follow-up time
							Field	Details	
Ali et al. (2016)	US	Retrospective, multi-centre, cohort study (ACS NSQIP, 2006–2010)	Spine surgery	294; NR	mFI-11	0 (non-frail), 0.09, 0.18, and $\ge$0.27 (severely frail)	CO	• General and surgical complications	1 month
								• Mortality	
Brown et al. (2020)	US	Retrospective, single-centre, cohort study (2013–2018)	Spine surgery for ASD	79; 51 ± 6.8	ASD-FI	< 0.3 (non-frail), $\ge$0.3 and < 0.5 (frail), and $\ge$0.5 (severely frail)	CO	• Costs and radiographic imaging	2 years
							PRO	• QALY calculated by the EQ-5D and ODI	
Chan et al. (2021)	US	Retrospective, multi-centre, cohort study (ACS NSQIP, 2010–2018)	Spine surgery (posterior approach) for degenerative lumbar spondylolisthesis	15,658; 62.5 ± 12.1 ^a^	mFI-5	0 (non-frail), 1 (prefrail), 2 (frail), and ≥ 3 (severely frail)	CO	• Clavien–Dindo grade IV complications	1 month
								• Non-home discharge, readmission, and reoperation	
Chang et al. (2020)	Korea	Prospective, single-centre, cohort study (2015–2018)	Elective surgery for lumbar spinal stenosis	261; 72.3 ± 4.8 ^a^	CGA	0–2 (non-frail) and ≥ 3 (frail)	CO	• General and surgical complications	1 month
					mFI-5	0–1 (non-frail) and ≥ 2 (frail)			
					mFI-11	< 0.27 (non-frail) and $\ge$0.27 (frail)			
Charest-Morin et al. (2018)	Canada	Ambispective, single-centre, cohort study (2009–2013)	Elective thoraco-lumbar surgery for DSD	102; median: 72, range: 68–78	mFI-11	0 (non-frail), > 0 and < 0.21 (prefrail), and $\ge$0.21(frail)	CO	• General and surgical complications and mortality	In hospital
								• LOS and non-home discharge	
Elsamadicy et al. (2021)	US	Retrospective, multi-centre, cohort study (ACS NSQIP, 2010–2016)	Spinal decompression and fusion	5,296; 61.2 ± 11.7 ^a^	mFI-5	0 (non-frail), 1 (mildly frail), and $\ge$2 (moderately to severely frail)	CO	• General and surgical complications	1 month
								• LOS, reoperation, and readmission	
Flexman et al. (2016)	US	Retrospective, multi-centre, cohort study (ACS NSQIP, 2006–2012)	Degenerative spine surgery	52,671; 56.1 ± 14.5	mFI-11	0 (non-frail), 0 > and < 0.21 (prefrail), and $\ge$0.21 (frail)	CO	• Clavien-Dindo grade 2 or higher complications, mortality	1 month
								• LOS and non-home discharge	
Hannah et al. (2020)	US	Retrospective, single-centre, cohort study (2008–2016)	Spine surgery for degenerative conditions	11,754; 54.6 ± 15.5 ^a^	HFRS	0–5 (low frailty), 6–15 (medium frailty), and $\ge$16 (high frailty)	CO	• Total complications	In hospital
								• ICU admission, LOS, and non-home discharge	
								• Costs	
								• Readmission and visit to the emergency room	1 and 3 months
Jung et al. (2022)	Korea	Retrospective, single-centre, cohort study (2012–2018)	LLIF	152; 66.0 ± 7.9 ^a^	mFI-11	0, 0.09, 0.18, and $\ge$0.27	CO	• General and surgical complications	In hospital
								• LOS, reoperation, and length of bed rest	
							PRO	• VAS for back and leg pain; ODI; substantial clinical benefit by VAS-B, VAS-L, and ODI	1 and 2 years
Kang et al. (2020)	Korea	Retrospective, single-centre, cohort study (2014–2018)	Simple and complex lumbar spinal fusion	584; 64.8 ± 13.8	mFI-5	0, 1, and $\ge$2	CO	• General and surgical complications	1 month
Kim et al. (2020)	Korea	Retrospective, single-centre, cohort study (2011–2016)	Thoracolumbar and lumbar (T9–S1) instrumentation spine surgery	138; 78.3 ± 2.8	mFI-11	0 (non-frail), 0 > and < 0.27 (prefrail), and $\ge$0.27 (frail)	CO	• General and surgical complications and mortality	6 months
								• Reintubation	
Kim et al. (2021)	Korea	Retrospective, single-centre, cohort study (2019–2020)	Elective thoracic or lumbar spine surgery	85; 74.1 ± 6.5	K-FRAIL	0 (robust), 1–2 (prefrail), and $\ge$3 (frail)	CO	• Postoperative complication	In hospital
								• LOS	
Leven et al. (2016)	US	Retrospective, multi-centre, cohort study (ACS NSQIP, 2005–2012)	Surgery for ASD	1,001; 59 ± 14	mFI-11	0 (non-frail), 0.09, 0.18, 0.27, and 0.36	CO	• General and surgical complications and mortality	1 month
								• Blood transfusion and reoperation	
Li et al. (2021)	China	Retrospective, single-centre, cohort study (2014–2017)	Long-segment corrective surgery for ASD (posterior approach)	161; 66.3 ± 8.5	mFI-11	< 0.27 (non-frail) and $\ge$0.27(frail)	CO	• Major complications by Glassman et al.	In hospital
								• Radiographic imaging	2 years
							PRO	• ODI, SRS-22, JOA, and VAS for back pain	
Miller et al. (2017)	US	Retrospective, multi-centre, cohort study (ISSG, 2010–2014)	Surgery for ASD	417; 56.6 ± 1.1 ^a^	ASD-FI	< 0.3 (non-frail), 0.3–0.5 (frail), and > 0.5 (severely frail)	CO	• Surgical complications and major complications by Glassman et al.	2 years
								• LOS and reoperation	
Miller et al. (2018, Jan)	US	Retrospective, multi-centre, cohort study (ISSG, 2009–2015)	Spine surgery for CD	61; 61.0 ± 2.4 ^a^	CD-FI	< 0.2 (non-frail), 0.2–0.4 (frail), and > 0.4 (severely frail)	CO	• General and surgical complications and major complications by Glassman et al.	1 year
								• ICU admission, LOS, and non-home discharge	
Miller et al. (2018, Sep)	Europe	Retrospective, multi-centre, cohort study (ESSG, 2012–2014)	Surgery for ASD	266; 50.7 ± 1.9 ^a^	ASD-FI	< 0.3 (non-frail), 0.3–0.5 (frail), and > 0.5 (severely frail)	CO	• General and surgical complications and major complications by Glassman et al. and McDonnel et al.	2 years
								• LOS and reoperation	
Miller et al. (2018, Oct)	Europe, Asia, and North America	Prospective, multi-centre, cohort study (Scoli-RISK-1, 2009–2011)	More invasive spine surgery for severe ASD	267; 57 ± 15	ASD-FI	< 0.3 (non-frail), 0.3–0.5 (frail), and > 0.5 (severely frail)	CO	• Major complications by Glassman et al. and McDonnel et al.	In hospital
								• LOS	
Passias et al. (2019)	US	Retrospective, multi-centre, cohort study (2013–2017)	Surgery for CD	121; 61.1 ± 10.1 ^a^	mCD-FI	< 0.3 (non-frail), 0.3–0.5 (frail), and > 0.5 (severely frail)	CO	• General and surgical complications and mortality	No data
								• LOS, non-home discharge, and reoperation	
							PRO	• NDI, NRS scores for back and neck pain, and EQ-5D scores	
Passias et al. (2022)	US	Retrospective, single-centre, cohort study (2014–2018)	Corrective surgery for ASD	560; 59 ± NR	mASD-FI	< 7 (non-frail), 7–12 (frail), and $>$12 (severely frail)	CO	• All complications	2 years
								• LOS and reoperation	
							PRO	• ODI, SRS-22 scores, EQ-5D scores, pain catastrophizing scale scores	
Phan et al. (2017)	US	Retrospective, multi-centre, cohort study (ACS NSQIP, 2010–2014)	ALIF	3,920; NR	mFI-11	0 (non-frail), 0.09, 0.18, $\ge$0.27	CO	• General and surgical complications and mortality	1 month
								• Blood transfusion, LOS > 5 days, and reoperation	
Pierce et al. (2020)	US	Retrospective, multi-centre, cohort study (ISSG, 2008–2018)	Spine fusion for ASD	191; 59 ± 12	ASD-FI	0 (non-frail), 0.3–0.5 (mildly frail), and > 0.5 (severely frail)	CO	• Major and minor complications and general and surgical complications	1 and 3 years
								• LOS	
							PRO	• NRS scores for back and leg pain, ODI, and SRS-22 scores	
Pierce et al. (2021)	US	Retrospective, multi-centre, cohort study (ISSG, 2013–2018)	Spine surgery for CD	106; 61.7 ± NR	mCD-FI	< 0.3 (non-frail) and $\ge$0.3 (frail)	CO	• Surgical and major complications	3 months and 1 year
								• Reoperation	
								• Radiographic imaging	
							PRO	• NDI, NRS scores for neck pain, EQ-5D scores, and mJOA scores	
Pierce et al. (2021)	US	Retrospective, multi-centre, cohort study (ACS NSQIP, 2005–2016)	Elective spine surgery	234,738; 57 ± NR	mFI-5	0 (non-frail), 0.3–0.5 (mildly frail), and > 0.5 (severely frail)	CO	• General and surgical complications and mortality	1 month
					mFI-11	-		• Readmission	
Pierce et al. (2021)	US	Retrospective, multi-centre, cohort study (ACS NSQIP, 2005–2016)	Elective surgery for ASD	9,143; 59.1 ± NR	mFI-5	0 (non-frail), 0.3–0.5 (mildly frail), and > 0.5 (severely frail)	CO	• General and surgical complications	1 month
								• LOS	
Pulido et al. (2022)	Europe	Retrospective, single-centre, cohort study (2011–2019)	Spine surgery	2,042; 60 ± 17	HFRS	0–5 (low frailty), 6–15 (medium frailty), and $\ge$16 (high frailty)	CO	• General and surgical complications	In hospital
								• Transfusion rate	
								• Reoperation	3 months
Reid et al. (2018)	US	Retrospective, multi-centre, cohort study (NR)	Fusion of $\ge$level 4 for ASD	332; 56.7 ± 14.8	ASD-FI	0 (non-frail), 0.3–0.5 (frail), and > 0.5 (severely frail)	PRO	• ODI, SF-36 PCS scores, NRS scores for back and leg pain	2 years
								• Substantial clinical benefit by Glassman et al.	
Rothrock et al. (2019)	US	Prospective, single-centre, cohort study (2013–2014)	Elective cervical or lumbar spine surgery	87; median: 71, range: 67–76	Phenotype	0 (non-frail), 1 or 2 (prefrail), and ≥ 3 (frail)	CO	• Frailty status (phenotype)	3 months
							PRO	• PQRS scores (for cognitive recovery and ADL)	1 and 3 days and 1 and 3 months
								• IADL (Alzheimer’s disease research centre)	1 and 3 months
Shahrestani et al. (2021)	US	Retrospective, multi-centre, cohort study (HCUP NRD, 2016–2017)	Single-level lumbar fusion	11,845; 72.5 ± 5.9 ^a^	JHACG	Grouping of non-frail and frail patients according to the JHACG frailty-defining diagnosis indicator	CO	• General complications	In hospital
								• LOS and non-home discharge	
								• Costs	
								• General and surgical complications at readmission	1 and 3 months
								• Mortality	1, 3, and 6 months
								• Readmission	
Shin et al. (2017)	US	Retrospective, multi-centre, cohort study (ACS NSQIP, 2005–2012)	ACDF or PCF	6,965; 53.71 ^a^ ± NR	mFI-11	0 (non-frail), 0.09, 0.18, 0.27, and $\ge$0.36	CO	• General and surgical complications	1 month
								• Mortality	
Sun et al. (2020)	China	Retrospective, single-centre, cohort study (2016–2018)	Elective posterior thoracolumbar fusion	426; 72.7 ^a^ ± NR	mFI-11	0 (non-frail), < 0.21 (prefrail), and $\ge$0.21 (frail)	CO	• General and surgical complications and major and minor complications	In hospital
								• LOS and non-home discharge	
								• Reoperation	1 month
								• Mortality	1 year
								• Readmission	
							PRO	• ODI and SF-36 scores	1 year
Susano et al. (2020)	US	Prospective, single-centre, cohort study (2017–2018)	Elective spine surgery	439; median: 75, range: 73–79	Phenotype	0 (non-frail), 1 or 2 (prefrail), and ≥ 3 (frail)	CO	• Delirium and all other-cause complications	In hospital
								• LOS and non-home discharge	
Ton et al. (2022)	US	Retrospective, multi-centre, cohort study (HCUP NRD, 2016–2017)	Multi-level lumbar fusion	7,088; 74.0 ± 5.8	JHACG	Grouping of non-frail and frail patients according to the JHACG frailty-defining diagnosis indicator		• Surgical and general complications	1, 3, or 6 months
								• Non-home discharge	
								• Costs	
Weaver et al. (2019)	US	Retrospective, multi-centre, cohort study (ACS NSQIP, 2012–2016)	Elective 1- or 2-level posterior lumbar fusion for degenerative lumbar pathology	23,516; NR	mFI-5	0, 1, and $\ge$2	CO	• Surgical and general complications and mortality	1 month
								• Blood transfusion, non-home discharge, readmission, reintubation, and postoperative ventilator use	
Wilson et al. (2020)	US	Retrospective, multi-centre, cohort study (ACS NSQIP, 2010–2018)	Cervical decompression and fusion including anterior and/or posterior approach	41,369; 56.6 ± NR	mFI-5	0 (non-frail), 1 (prefrail), 2 (frail), ≥ 3 (severely frail)	CO	• Major complications and mortality	1 month
								• LOS, non-home discharge, readmission, and reoperation	
					mFI-11	0 (non-frail), 0.09 (prefrail), 0.18 (frail), and $\ge$0.27 (severely frail)			
Yagi et al. (2018)	Japan	Retrospective, multi-centre, cohort study (NR)	At least a 5-level elective spine surgery for ADS, DS, and LSCS	481; 66.9 ± 9.4 ^a^	mFI-11	0 (non-frail), < 0.21 (prefrail), and $\ge$0.21(frail)	CO	• Total complication rates	2 years
								• Radiographic imaging	6 weeks and 2 years
							PRO	• ODI, SF-36 scores, SRS-22 scores, and VAS scores for low back and leg pain	2 years
Yagi et al. (2019)	Japan	Retrospective, multi-centre, cohort study (NR)	Spine surgery for ASD	281; 54.4 ± 18.7	mFI-5	0 (robust), 1 (prefrail), and $\ge$2 (frail)	CO	• General, surgical, and major complications	2 years
					mFI-11	0 (robust), < 0.27 (prefrail), and $\ge$0.27 (frail)		• SAEs (Clavien–Dindo grade $\ge$3, reoperation required, deterioration of motor function at discharge, or new motor deficit)	
Zreik et al. (2021)	US	Retrospective, multi-centre, cohort study (ACS NSQIP, 2016–2018)	Elective ACDF for degenerative disease	23,754; median: 55, range: 47–63	mFI-5	0, 1, and $\ge$ 2	CO	• Minor and major complications	1 month
								• LOS, non-home discharge, and readmission	

Age is presented as mean and standard deviation or median and interquartile range

Abbreviation: ACS NSQIP, American College of Surgeons National Surgical Quality Improvement Program; NR, not reported; mFI-5, 5-item modified frailty index; mFI-11, 11-item modified frailty index; ASD, adult spinal deformity; ASD-FI, adult spinal deformity-frailty index; QALY, Quality-adjusted life years; EQ-5D, EuroQol-5D; ODI, Oswestry disability index; CGA, comprehensive geriatric assessment; CO, clinical outcome; DSD, degenerative spinal disease; LOS, length of hospital stay; HFRS, Hospital Frailty Risk Score; ICU, intensive care unit; LLIF, lateral lumbar interbody fusion; VAS, visual analogue scale; K-FRAIL, Korean version of the fatigue, resistance, ambulation, illnesses, and loss of weight; CD, cervical spine deformity; CD-FI, cervical spine deformity-frailty index; ISSG, international spine study group; ESSG, European spine study group; NDI, Neck Disability Index; NRS, numerical rating scale; mAS-FI, modified adult spinal deformity frailty index; ALIF, anterior lumbar interbody fusion; SRS-22, Scoliosis Research Society 22-question; JOA, Japanese orthopedic association scale; SF-36, 36-Item Short Form Survey; PCS, physical component summary; PQRS, Postoperative Quality of Recovery Scale; PRO, patient-reported outcome; ADL, activities of daily living; IADL, instrumental activities of daily living; HCUP NRD, Healthcare Cost and Utilization Project Nationwide Readmission Database; JHACG, Johns Hopkins Adjusted Clinical Groups; ACDF, anterior cervical discectomy and fusion; ADS, adult degenerative scoliosis; DS, degenerative spondylolisthesis; LSCS, lumbar spinal canal stenosis; PCF, posterior cervical fusion; SAEs, severe adverse event

### Clinical outcomes

All studies, except one [47], considered COs as postoperative health-related outcomes. The COs included postoperative complications and supportive management procedures.

In 35 studies, the postoperative complications were addressed as COs [7, 10–12, 14, 16, 22–24, 29–46, 49–56]. The postoperative complications were further divided into general and surgical complications. The general complications comprised anaemia; electrolyte abnormalities; cardiovascular, gastrointestinal, pulmonary, renal, and urinary complications; delirium; deep vein thrombosis; falls; and sepsis/septic shock. The surgical complications comprised dural tears, excessive bleeding, hematomas, instrumentation failure, neurological symptoms, positional and wound-related complications, pseudoarthrosis, pneumoperitoneum, and kyphosis. These complications were classified as minor or major or I–IV (Clavien–Dindo classification) [57]. In five studies [16, 22, 37–39], the definition provided by Glassman et al. was used to determine the major complications [58, 59]. In 13 studies [10, 12, 23, 24, 29, 32, 35, 41, 44, 49, 50, 52, 53], mortality was considered a postoperative complication.

Supportive management procedures included transfusion for bleeding [10, 41, 46, 52], admissions to intensive care units [14, 22], length of hospital stay [11, 12, 14, 22, 24, 29, 31–33, 36–42, 45, 50, 53, 56], length of bed rest [33], nonhome discharge [7, 11, 12, 14, 22, 24, 29, 32, 50–53, 56], postoperative ventilator use [52], reintubation [35, 52, 56], readmission [7, 14, 24, 31, 44, 50, 52, 53, 56], reoperation [7, 10, 29, 31, 33, 37, 38, 40, 41, 43, 46, 50, 53], and emergency room visit [14].

Other COs included costs [13, 14, 24, 51], frailty status [48], and radiographic imaging findings [13, 16, 43, 54].

### Patient reported outcomes

Eleven studies assessed PROs [13, 16, 29, 33, 40, 42, 43, 47, 48, 50, 54]. The PROs were assessed using the instrumental activities of daily living [48], EuroQol-5D (EQ-5D) [13, 29, 40], Japanese Orthopaedic Association (JOA) score [16], modified mJOA score [43], Neck Disability Index [29, 43], Oswestry Disability Questionnaire (ODI) [13, 16, 33, 40, 42, 47, 50, 54], numerical rating scale for pain [29, 42, 43, 47], Postoperative Quality of Recovery Scale for cognitive recovery and activities of daily living [48], Pain Catastrophizing Scale [40], 36-Item Short Form Survey (SF-36) [47, 50, 54], Scoliosis Research Society 22-question [16, 40, 42, 54], and visual analogue scale for pain [16, 33, 54].

Substantial clinical benefit was determined based on changes in the ODI, SF-36 score, and back and leg pain score after the surgery [33, 47]. The quality-adjusted life years were determined using the EQ-5D [13].

### Meta-analysis of the selected outcomes

#### Synthesis of meta-analysis results regarding the clinical outcomes

**Fig. 3:**
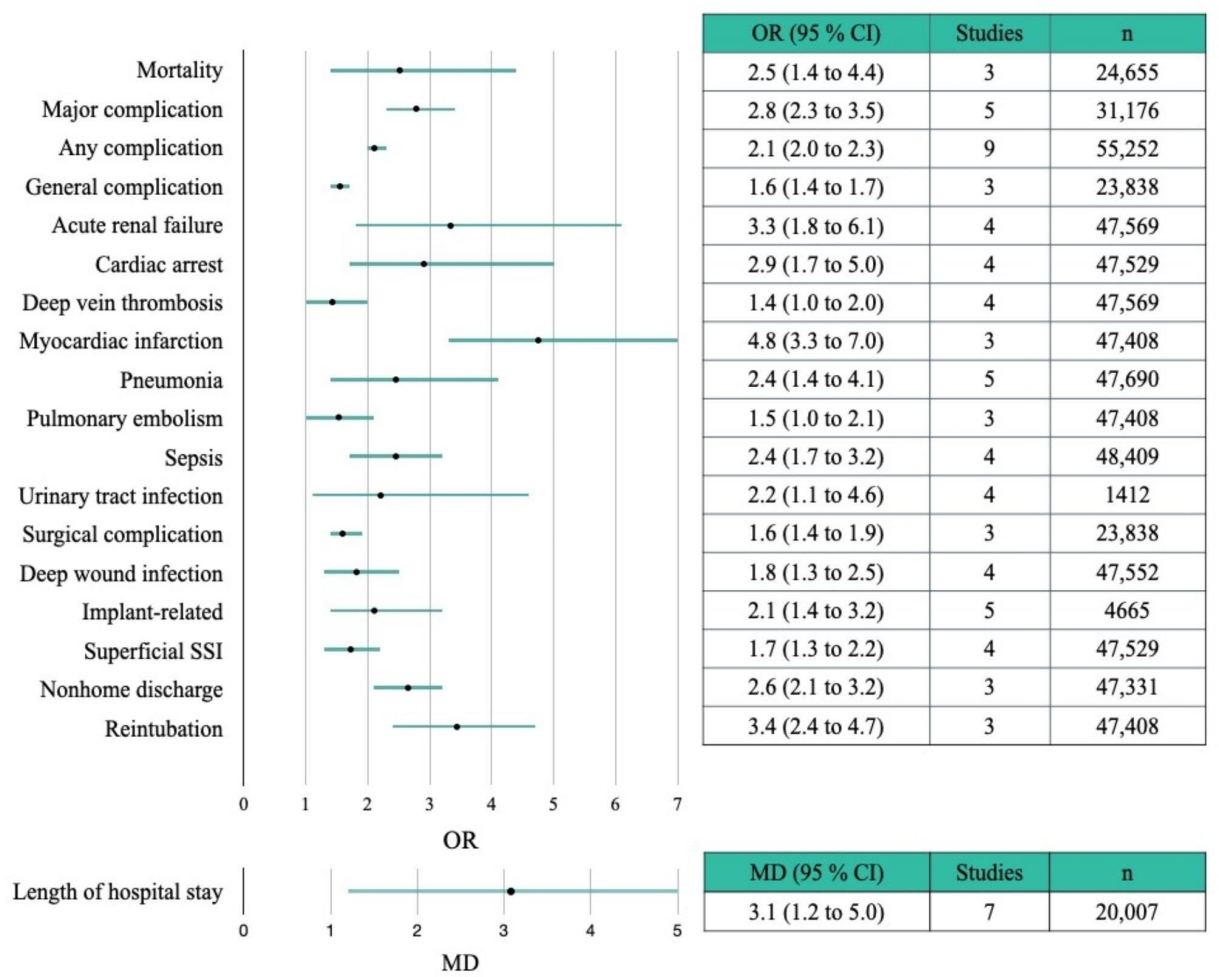
Forest plots of the clinical outcomes that showed significant results in the meta-analysis. SSI, surgical site infection; OR, odds ratio; MD, mean difference; CI, confidence interval

Results of the meta-analysis of the COs are presented in Table 2. A forest plot depicting significant associations between COs and frailty is shown in Fig. 3. Compared to the non-frail group, the frail group was more likely to experience the following COs: mortality (OR = 2.5; 95% CI = 1.4–4.4) [10, 35, 52], major complication (OR = 2.8; 95% CI = 2.3–3.5) [39, 42, 49, 56], any complication (OR = 2.1; 95% CI = 2.0–2.3) [10, 29, 35, 39, 40, 42, 52, 55, 56], general complication (OR = 1.6; 95% CI = 1.4–1.7) [22, 30, 52], acute renal failure (OR = 3.3; 95% CI = 1.8–6.1) [16, 35, 52, 56], cardiac arrest (OR = 2.9; 95% CI = 1.7–5.0) [29, 35, 52, 56], deep vein thrombosis (OR = 1.4; 95% CI = 1.0–2.0) [16, 35, 52, 56], gastrointestinal complication (OR = 0.9; 95% CI = 0.4–1.9) [16, 29, 33, 42], myocardial infarction (OR = 4.8; 95% CI = 3.3–7.0) [35, 52, 56], pneumonia (OR = 2.4; 95% CI = 1.4–4.1) [16, 29, 35, 52, 56], pulmonary embolism (OR = 1.5; 95% CI = 1.0–2.1) [35, 52, 56], sepsis (OR = 2.4; 95% CI = 1.7–3.2) [10, 35, 52, 56], stroke/cerebrovascular accident (OR = 2.1; 95% CI = 0.5–8.5) [16, 35, 41], urinary tract infection (OR = 2.2; 95% CI = 1.1–4.6) [10, 29, 33, 35], surgical complication (OR = 1.6; 95% CI = 1.4–1.9) [22, 30, 52], deep wound infection (OR = 1.8; 95% CI = 1.3–2.5) [16, 29, 52, 56], implant-related complication (OR = 2.1; 95% CI = 1.4–3.2) [29, 33, 41, 42, 55], neurological complication (OR = 1.1; 95% CI = 0.6–1.7) [16, 29, 33, 41, 42], superficial surgical site infection (OR = 1.7; 95% CI = 1.3–2.2) [29, 35, 52, 56], length of stay (MD = 3.1; 95% CI = 1.2–5.0) [13, 16, 24, 33, 37, 38, 51], non-home discharge (OR = 2.6; 95% CI = 2.1–3.2) [22, 52, 56], reintubation (OR = 3.4; 95% CI = 2.4–4.7) [35, 52, 56], and reoperation (OR = 1.0; 95% CI = 0.4–2.5) [10, 29, 33, 52]. The forest plot for each CO is presented in Supplementary Material Fig. 2.

**Table 2:** Meta-analysis of the health-related outcomes in terms of the preoperative frailty status

Outcomes			Studies	Statistical Method	OR or MD or SMD [95% CI]	*P* value	I^2^ (%)
Clinical outcomes							
	Mortality		3	M–H, Fixed	2.5 [1.4; 4.4]	0.002	20
	Major complication		5	M–H, Fixed	2.8 [2.3; 3.5]	< 0.001	46
	Any complication		9	M–H, Random	2.1 [2.0; 2.3]	< 0.001	63
	General complication		3	M–H, Fixed	1.6 [1.4; 1.7]	< 0.001	36
		Acute renal failure	4	M–H, Fixed	3.3 [1.8; 6.1]	< 0.001	14
		Cardiac arrest	4	M–H, Fixed	2.9 [1.7; 5.0]	< 0.001	0
		Deep vein thrombosis	4	M–H, Fixed	1.4 [1.0; 2.0]	0.033	31
		Gastrointestinal complication	4	M–H, Fixed	0.9 [0.4, 1.9]	0.767	0
		Myocardial infarction	3	M–H, Fixed	4.8 [3.3; 7.0]	< 0.001	0
		Pneumonia	5	M–H, Random	2.4 [1.4; 4.1]	< 0.001	52
		Pulmonary embolism	3	M–H, Fixed	1.5 [1.0; 2.1]	0.039	0
		Sepsis	4	M–H, Fixed	2.4 [1.7; 3.2]	< 0.001	42
		Stroke/CVA	3	M–H, Fixed	2.1[0.5; 8.5]	0.314	15
		Urinary tract infection	4	M–H, Fixed	2.2 [1.1; 4.6]	0.027	0
	Surgical complication		3	M–H, Fixed	1.6 [1.4; 1.9]	< 0.001	21
		Deep wound infection	4	M–H, Fixed	1.8 [1.3; 2.5]	< 0.001	0
		Implant-related complication	5	M–H, Fixed	2.1 [1.4; 3.2]	< 0.001	40
		Neurological complication	5	M–H, Fixed	1.1 [0.6; 1.7]	0.821	29
		Superficial SSI	4	M–H, Fixed	1.7 [1.3; 2.2]	< 0.001	25
	Supportive management strategy						
		Length of hospital stay	7	IV, Random	3.1 [1.2; 5.0]	0.002	99
		Non-home discharge	3	M–H, Random	2.6 [2.1; 3.2]	< 0.001	76
		Reintubation	3	M–H, Fixed	3.4 [2.4; 4.7]	< 0.001	0
		Reoperation	4	M–H, Random	1.0 [0.4; 2.5]	1.000	64
Patient-reported outcome							
		Changes in the ODI	3	IV, Random	-9.6 [-23.0; 3.8]	0.151	90

Abbreviation: OR, odds ratio; MD, mean difference; M–H, Mantel–Haenzel; IV, inverse variance; CVA, cerebrovascular accident; SSI, surgical site infection; ODI, Oswestry disability index

The incidence rates of complications in the frail group and the robust group are presented in Supplementary Table 3. In the robust group, the five most prevalent complications, in descending order, were as follows: gastrointestinal complications (5.6%), urinary tract infection (4.6%), implant-related complications (1.5%), neurological complications (1.4%), and superficial surgical site infections (0.6%). In contrast, in the frail group, the five most prevalent complications, in descending order, were as follows: implant-related complications (21.5%), neurological complications (13.6%), urinary tract infections (9.3%), gastrointestinal complications (5.6%), and stroke/cerebrovascular accidents (2.1%).

### Synthesis of meta-analysis results regarding the patient-reported outcomes

Results of the meta-analysis of the PROs are presented in Table 2. A forest plot for the PROs is shown in Supplementary Material Fig. 3. Changes in the ODI scores between pre- and post-surgery, categorized by frailty, were synthesized based on three papers [13, 16, 47]. The changes between pre- and post-operative ODI scores were not associated with preoperative frailty (MD= -9.6, 95% CI= -23–3.8).

### Sensitivity analysis

A sensitivity analysis was performed to identify the relationship between any complication and frailty, which had the highest number of synthesized papers. As shown in the forest plot for any complication (Supplementary Material Figs. 2 and 3), it was judged that heterogeneity occurred due to the articles by Passias et al. [29] and Kim et al. [35]. When a meta-analysis was performed by removing those two articles, the I^2^ value was reduced to 53% and 47%, respectively (Supplementary Material Fig. 4). Therefore, after removing these two papers, the meta-analysis was performed again (Supplementary Material Fig. 5). A fixed-effect model was selected because the heterogeneity was reduced to 10% for I^2^. The OR for any complication was 2.1 (95% CI = 2.0–2.3), which did not differ significantly from the original OR of 2.1. The findings of the sensitivity analysis indicate that the results of this study are reliable.

## Discussion

This systematic review and meta-analysis examined the association between preoperative frailty and postoperative health-related outcomes in patients who underwent spine surgery for degenerative spinal disease. In the 38 included studies, 10 frailty instruments were used to measure preoperative frailty and two typologies of health-related outcomes for the preoperative frailty status were identified. Preoperative frailty was observed to be associated with postoperative adverse health-related outcomes. It increased the incidence of adverse COs, including mortality and complications, but there was no significant difference with respect to the improvement of the postoperative PROs.

Research on frailty has increased appreciably recently; this includes studies on preoperative frailty and its association with COs [15, 60] or PROs [61] and studies on the construct validity of frailty instruments [62]. Previous studies conducted in surgical settings highlight the important role of frailty as a prognostic factor for considering surgery [15, 60, 61, 63]. A systematic review and meta-analysis of 19 studies on patients undergoing cardiac surgery revealed that frailty was associated with a two-fold greater risk of mortality, greater complications, and five-fold greater risk of non-home discharge [60]. In another systematic review and meta-analysis of 71 studies on adult patients undergoing cancer surgery, frailty was found to be related to a three-fold, two-fold, and four-fold greater risk of 30-day mortality, postoperative complications, and long-term mortality, respectively [15]. Our findings corroborate and extend the existing evidence on the association of preoperative frailty with postoperative adverse COs.

Factors other than age should be considered when predicting postoperative recovery in patients with degenerative spinal diseases [17, 20]. The prevalence of frailty is increasing among individuals undergoing spine surgeries. Analysis of a patient population that underwent spine surgery, using data from the American College of Surgeons National Surgical Quality Improvement Program database, revealed that the number of frail patients doubled from 2005 to 2016 [44]. This suggests that frailty is an important variable to consider for risk stratification when predicting postoperative recovery in patients with degenerative spinal disease [17, 20]. The frailty score may serve as a preoperative screening tool to aid in decision-making and perioperative management. It can help monitor patients’ health, thereby allowing healthcare professionals to identify high-risk patients and develop better treatment strategies. It can also help guide discussions among healthcare professionals, patients, and family members to reduce surgical vulnerability, enable pre-habilitation to increase patient resilience, and customize perioperative care [64, 65].

In our qualitative synthesis, clinical outcomes were identified as health-related outcomes in all but one study [47]. Postoperative complications can be divided into general and surgery-related complications. Supportive management strategies include blood transfusions and unplanned intubations; these represent additional supportive care provided to patients with problems that are not part of the normal recovery process.

Among the COs in this study, 19 items were synthesized for quantitative analysis, and 3–9 studies participated in the synthesis. If there are fewer than 10 studies, statistical confirmatory tests for publication bias (e.g. the funnel test) are not recommended [25]; thus, publication bias could not be confirmed in this study. Therefore, items that showed heterogeneity, such as any complications, pneumonia, length of hospital stay, non-home discharge, and reoperation, should be interpreted carefully. In case of any complications, a sensitivity analysis was performed because the number of studies was considerably large and heterogeneity was noted across the studies. This analysis identified two studies as outliers [29, 35], and the synthesis was attempted again by excluding them. The re-analysis revealed that the heterogeneity improved and the effect size did not affect the existing results.

The meta-analysis of the clinical outcomes in this study revealed that the risk of mortality in the frail group was 2.5 times higher than that in the non-frail group. Furthermore, the probability of major complication, any complication, general complication, acute renal failure, cardiac arrest, deep vein thrombosis, myocardial infarction, pneumonia, pulmonary embolism, sepsis, stroke/cerebrovascular accident, surgical complication, deep-wound infection, implant-related complication, superficial surgical site infection, length of hospital stay, nonhome discharge, and reintubation was higher in the frail group than in the non-frail group. Notably, the order of complication prevalence was different between the robust and frail groups. In the robust group, the most common complication was relatively simple gastrointestinal complications, while in the frail group, relatively severe implant-related complications, which might necessitate reoperation, were the most common. The increased incidence of complications or the severity of complications in frail patients can be attributed to several factors. Frailty is linked to reduced immune function, which can result in compromised ability to cope with complications such as infections during the stress of post-surgery recovery [66]. Frailty is associated with decreased metabolic activity, such as high levels of glucose and LDL cholesterol, which can impair tissue nutrient supply and metabolic functions [67], ultimately hindering post-surgery recovery capacity. Furthermore, frailty is associated with low physical activity levels and reduced muscle mass [66, 68], which might persist post-surgery, leading to compromised recovery due to limited physical activity. Healthcare professionals who deliver postoperative care to frail patients should be aware of these complications. This can lead to increases in the time of direct nursing care and the cost of physical resources such as ICU and rehabilitation, as well as convalescent care beds [69].

Another key knowledge gap that thwarts a more meaningful prognosis is the lack of data on PROs. Studies have paid considerable attention to frailty as an important preoperative risk indicator for COs [15, 61]; similar studies for PROs are few. Data on cognitive outcomes, functional outcomes, and quality of life are lacking. In our systematic review, only 11 of 38 studies reported the effects of frailty on the PROs (e.g., quality of life, ODI, and pain); the multidimensional health status of patients was reported in just six studies [13, 29, 40, 47, 50, 54]. The wide variety of outcome measures limited the comparison of results among the included studies. The meta-analysis revealed that frailty was not significantly associated with the postoperative ODI and changes in the perioperative ODI; however, it had a conflicting relationship with the COs. Specifically, compared to non-frail patients, frail patients experienced greater improvements in ODI, quality of life, and pain [47]. Such improvements are partly explained by corrections in postural deformity, as frail patients have worse preoperative sagittal imbalances than those who do not [70, 71]. When choosing the best treatment options for patients with degenerative spinal diseases, it is necessary to consider their preferences and values [72, 73]. Frailty assessment can help patients and their families make informed decisions before surgery. It highlights the need for future studies to determine the association between frailty and PROs in patients with degenerative spinal disease.

We identified the typologies of postoperative health-related outcomes associated with preoperative frailty in patients who underwent spine surgery for degenerative spinal disease. These typologies can inform the content and structure of pre-rehabilitation and customized educational programs for patients undergoing spine surgery. They can also be used as basic data for implementing programs or pathways to reverse frailty in patients with spinal diseases and improve their health-related outcomes. Furthermore, the identified typologies can help develop evaluation tools to evaluate frailty-associated health-related outcomes in patients undergoing spine and other surgeries.

Finally, frailty is an important prognostic marker for postoperative health-related outcomes in patients with degenerative spinal disease, but there is a lack of consensus on the best means to accurately and efficiently determine frailty in patients undergoing spine surgery. In this review and meta-analysis, 10 different frailty instruments (including the mFI-5, mFI-11, and ASD-FI) were used to define frailty, and the variability in the evaluations by the same tool was demonstrated. A review of 14 different tools used for the assessment of frailty in a population undergoing spine surgery (age: >18 years) revealed wide variabilities in the tool components, time required to complete the assessment, and efficacy of outcome prediction among the tools [74]. Furthermore, significant heterogeneity was observed among the tools with respect to the cut-off values for risk establishment and stratification. In acute care hospitals, it is difficult to determine the most suitable tool for clinical practice. Future studies must prospectively validate frailty tools to confirm their effectiveness and applicability as reliable risk-stratification tools for the diagnosis of frailty among patients with degenerative spinal disease.

This study has some limitations. First, a meta-analysis of some items could not be performed due to data heterogeneity. Specifically, although all patients underwent spine surgery, the severity of the surgery differed among the studies because of a mixture of fusion and decompression. Furthermore, the detection of COs differed due to a mixture of prospective and retrospective studies. There were inconsistencies among the studies in the definition of frailty and the scales used for frailty analysis. Furthermore, there was heterogeneity among the frailty tools used. Second, only less than half of the included studies were included in the meta-analyses due to insufficient data (e.g., some studies reported only comparing ratios; for the same patient in the same database, only the first studies published first were considered). Third, because there were few than 10 studies in our meta-analysis, we could not identify or evaluate publication bias.

The number of patients undergoing spine surgery for degenerative spinal diseases is increasing. Thus, despite the aforementioned limitations, our study is of high clinical value because it evaluated the effects of frailty on the health-related outcomes of these patients. Our findings can guide future studies and aid healthcare professionals who treat patients with degenerative spinal diseases.

## Conclusion

This systematic review and meta-analysis identified frailty as a strong predictor of COs in patients after spine surgery; however, preoperative frailty and PROs are still inconclusive. Further studies are needed to investigate the association between frailty and PROs. With the increasing number of frail patients undergoing spine surgery for degenerative spinal diseases, healthcare professionals should be aware of the effects of frailty and develop improved and focused perioperative management strategies for stratified frail patients. In particular, the development of interventions comprising treatment goals and plans that consider preoperative frailty as a risk factor for mortality and poor functional recovery can be an important cornerstone of preoperative management. Future research should focus on the development and implementation of interventions that could potentially improve postoperative cognitive, functional, and adverse outcomes in frail patients undergoing spine surgery.

### Electronic supplementary material

Below is the link to the electronic supplementary material.

Supplementary Material 1

## Data Availability

The original contributions presented in the study are included in the article/supplementary material, further inquiries can be directed to the corresponding author.

## Abbreviations

CO: Clinical outcomes
PRO: Patient-reported outcome
FI: Frailty index
mFI: Modified frailty index
ASD: Adult spinal deformity
CD: Cervical deformity
RoBANS: The Risk of Bias Assessment Tool for Nonrandomized Studies
OR: Odds ratio
CI: Confidence interval
MD: Mean difference
EQ-5D: EuroQol-5D
JOA: Japanese Orthopaedic Association
ODI: Oswestry Disability Questionnaire
SF-36: 36-Item Short Form Survey
