# Supplementary material for: Impact of frailty on the outcomes of patients undergoing degenerative spine surgery: a systematic review and meta-analysis
Source: BMC Geriatr. 2023 Nov 23;23:771. doi: 10.1186/s12877-023-04448-2 (PMC10668507; doi:10.1186/s12877-023-04448-2)
Supplement: Supplementary file 1 — Supplementary Material 1 [file 12877_2023_4448_MOESM1_ESM.docx]

**Supplementary Material Content**

| **Supplementary Material Table 1** | **Search strategies** | ∙∙∙∙∙∙∙∙∙∙∙∙∙∙∙∙∙∙∙∙∙∙∙∙∙∙∙∙∙∙∙∙∙∙∙∙∙∙∙∙∙∙∙∙∙∙∙∙∙∙∙∙∙∙∙∙∙∙∙∙∙∙∙∙∙∙∙∙∙∙∙∙∙∙∙∙∙∙∙∙∙∙∙ | | | | | | | | **2** |
| --- | --- | --- | --- | --- | --- | --- | --- | --- | --- | --- |
| **Supplementary Material Figure 1** | **Risk of bias in individual studies** | | ∙∙∙∙∙∙∙∙∙∙∙∙∙∙∙∙∙∙∙∙∙∙∙∙∙∙∙∙∙∙∙∙∙∙∙∙∙∙∙∙∙∙∙∙∙∙∙∙∙∙∙∙∙∙∙∙∙∙∙∙∙ | | | | | | | **4** |
| **Supplementary Material Table 2** | **Health-related outcome in each study** | | | ∙∙∙∙∙∙∙∙∙∙∙∙∙∙∙∙∙∙∙∙∙∙∙∙∙∙∙∙∙∙∙∙∙∙∙∙∙∙∙∙∙∙∙∙∙∙∙∙∙∙∙∙∙∙∙∙∙∙∙∙∙∙∙∙∙∙ | | | | | | **5** |
| **Supplementary Material Table 3** | **The rates of complication in the robust and frail group** | | | | | ∙∙∙∙∙∙∙∙∙∙∙∙∙∙∙∙∙∙∙∙∙∙∙ | | | | **13** |
| **Supplementary Material Figure 2** | **Forest plots for each clinical outcome** | | | | ∙∙∙∙∙∙∙∙∙∙∙∙∙∙∙∙∙∙∙∙∙∙∙∙∙∙∙∙∙∙∙∙∙∙∙∙∙∙∙∙∙∙∙∙∙∙∙∙∙∙∙∙∙∙∙∙∙∙∙∙∙ | | | | | **14** |
| **Supplementary Material Figure 3** | **Forest plots for the patient-reported outcome of the meta-analyses** ∙∙∙∙∙∙∙∙∙∙∙∙ | | | | | | | | ∙∙∙∙∙ | **20** |
| **Supplementary Material Figure 4** | **Forest plot after sensitivity analyses for any complication** | | | | | | ∙∙∙∙∙∙∙∙∙∙∙∙∙∙∙∙∙∙∙∙∙∙∙∙ | | | **21** |
| **Supplementary Material Figure 5** | **Forest plot for any complication after removing outlier studies** | | | | | | | ∙∙∙∙∙∙∙∙∙∙∙∙∙∙∙∙∙∙∙∙∙∙∙∙∙∙∙∙∙∙∙∙∙∙∙∙∙∙ | | **22** |

**Supplementary Material Table 1. Search strategies**

| No. | Search Term | Searched Article |
| --- | --- | --- |
| Ovid-MEDLINE | | |
| 1 | (Spine OR spinal OR vertebra* OR lumbar OR cervical OR thoracic).mp | 1,120,079 |
| 2 | (surgery or operati*).mp. | 3,507,915 |
| 3 | (anesthesia).mp. | 277,237 |
| 4 | (frail*).mp. | 35,777 |
| 5 | 2 OR 3 | 3,522,078 |
| 6 | 1 AND 4AND 5 | 606 |
| 7 | (“Postoperative period” OR postoperative OR post-operative OR “after surgery” OR “after operation”).mp. | 1,037,445 |
| 8 | (Outcome* OR effect* OR impact* OR morbidity OR mortality OR survival OR "length of stay" OR readmission OR adverse OR risk OR complication* OR prevalence OR recovery OR rehabilitation OR “quality of life” ).mp. | 16,919,903 |
| 9 | 7 AND 8 | 917,458 |
| 10 | 6 AND 9 | 332 |
| Ovid- EMBASE | | |
| 1 | (Spine OR spinal OR vertebra* OR lumbar OR cervical OR thoracic).mp | 1,333,231 |
| 2 | (surgery or operati*).mp. | 4,776,726 |
| 3 | (anesthesia).mp. | 410,424 |
| 4 | (frail*).mp. | 53,527 |
| 5 | 2 OR 3 | 4,982,452 |
| 6 | 1 AND 4AND 5 | 1,228 |
| 7 | (“Postoperative period” OR postoperative OR post-operative OR “after surgery” OR “after operation”).mp. | 1,377,216 |
| 8 | (Outcome* OR effect* OR impact* OR morbidity OR mortality OR survival OR "length of stay" OR readmission OR adverse OR risk OR complication* OR prevalence OR recovery OR rehabilitation OR “quality of life” ).mp. | 19,701,263 |
| 9 | 7 AND 8 | 1,197,733 |
| 10 | 6 AND 9 | 594 |
| CINAHL | | |
| 1 | (MH "Spine+") OR spine OR spinal OR (MH "Cervical Vertebrae+") OR (MH "Lumbar Vertebrae") OR (MH "Thoracic Vertebrae") OR vertebra OR vertebrae OR vertebral OR ((lumbar OR thoracic OR thoracolumbar OR cervical) AND (sclerosis OR deformity)) | 142,349 |
| 2 | (MH "Surgery, Operative+") OR surgery OR operati* | 1,047,307 |
| 3 | (MH "Anesthesia+") OR anesthesia | 79,647 |
| 4 | Frail* | 20,389 |
| 5 | 2 OR 3 | 1,078,990 |
| 6 | 1 AND 4AND 5 | 144 |
| 7 | (MM "Postoperative Period") OR “postoperative period” OR postoperative OR post-operative OR “after surgery” OR “after operation” | 219,466 |
| 8 | (MH "Outcomes (Health Care)+") OR outcome* OR effect* OR impact* OR (MM "Length of Stay") OR “length of stay” OR (MH "Morbidity+") OR morbidity OR (MH "Mortality") OR mortality OR (MM "Survival") OR survival OR (MM "Readmission") OR readmission OR adverse OR risk OR (MH "Postoperative Complications+") OR complication* OR (MM "Prevalence") OR prevalence OR (MH "Quality of Life+") OR “quality of life” OR (MH "Recovery+") OR recovery OR (MH "Rehabilitation+") OR rehabilitation | 3,945,810 |
| 9 | 7 AND 8 | 200,968 |
| 10 | 6 AND 9 | 85 |
| Cochrane Library | | |
| 1 | MeSH descriptor: [Spine] explode all trees OR spine OR spinal OR MeSH descriptor: [Cervical Vertebrae] explode all trees OR MeSH descriptor: [Lumbar Vertebrae] explode all trees OR MeSH descriptor: [Thoracic Vertebrae] explode all trees OR vertebrae OR vertebra OR vertebral OR ((lumbar OR thoracic OR thoracolumbar OR cervical) AND (deformity OR sclerosis)) | 48,877 |
| 2 | MeSH descriptor: [General Surgery] explode all trees OR surgery OR operati* | 305,902 |
| 3 | MeSH descriptor: [Anesthesia] explode all trees OR anesthesia | 88,835 |
| 4 | frail* | 4,883 |
| 5 | 2 OR 3 | 337,677 |
| 6 | 1 AND 4AND 5 | 64 |
| 7 | MeSH descriptor: [Postoperative Period] explode all trees OR “postoperative period” OR postoperative OR “post-operative” OR “after surgery” OR “after operation” | 150,791 |
| 8 | MeSH descriptor: [Critical Care Outcomes] explode all trees OR outcome* OR impact* OR effect* OR MeSH descriptor: [Length of Stay] explode all trees OR “length of stay” OR MeSH descriptor: [Morbidity] explode all trees OR morbidity OR MeSH descriptor: [Mortality] explode all trees OR mortality OR MeSH descriptor: [Survival] explode all trees OR survival OR MeSH descriptor: [Patient Readmission] explode all trees OR readmission OR MeSH descriptor: [Long Term Adverse Effects] explode all trees OR adverse OR MeSH descriptor: [Risk] explode all trees OR risk OR MeSH descriptor: [Postoperative Complications] explode all trees OR complication* OR MeSH descriptor: [Prevalence] explode all trees OR prevalence OR MeSH descriptor: [Quality of Life] explode all trees OR “quality of life” OR MeSH descriptor: [Recovery of Function] explode all trees OR recovery OR MeSH descriptor: [Rehabilitation] explode all trees OR rehabilitation | 1,493,409 |
| 9 | 7 AND 8 | 141,680 |
| 10 | 6 AND 9 | 36 |

**
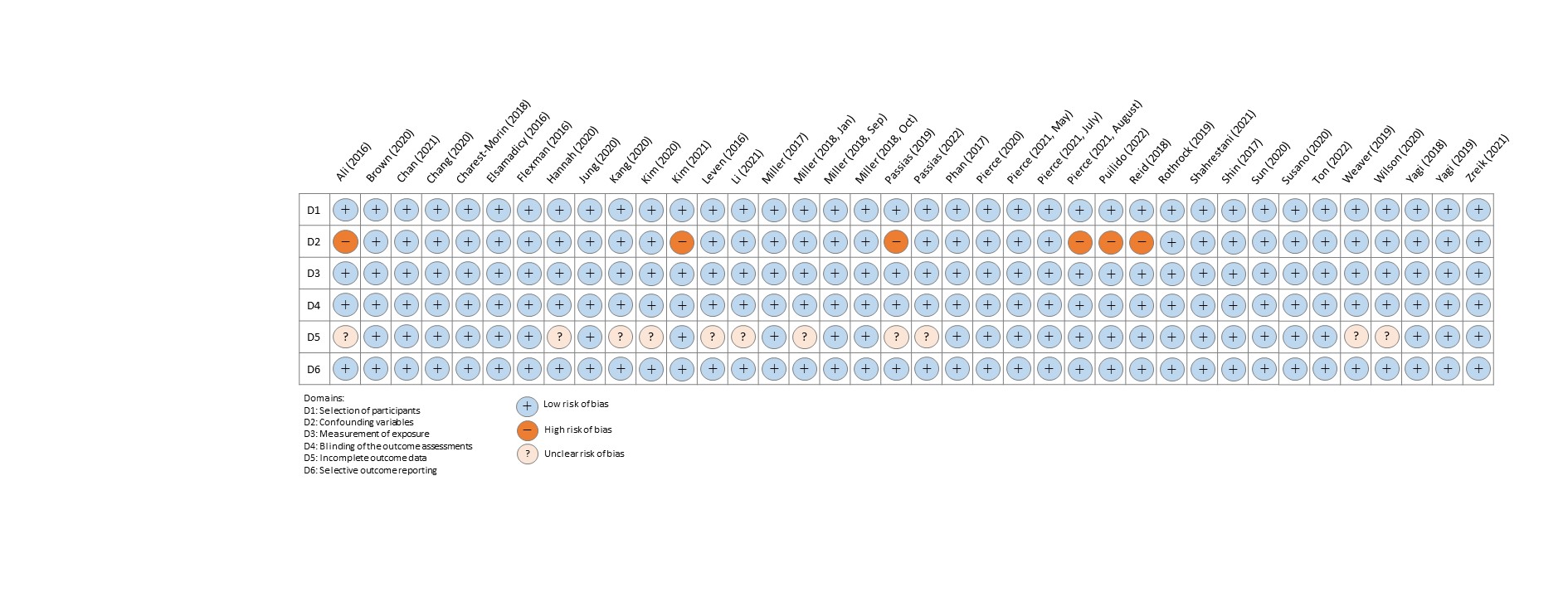
**

**Supplementary Material Figure 1. Risk of bias in individual studies**

**Supplementary Material Table 2. Health-related outcome in each study**

| Author (year) |  | Postoperative outcome |
| --- | --- | --- |
|  | Field | Detail |
| Ali et al. (2016) | CO | • Wound infection (surgical site infection, including superficial, deep, and organ space infections), any infection (wound infection, urinary tract infection, or pneumonia), Clavien-Dindo grade IV complication (pulmonary embolism, renal failure, prolonged ventilator care, and stroke) |
|  |  | • Mortality |
| Brown et al. (2020) | CO | • Cost |
|  |  | • Radiographic imaging |
|  | PRO | • Quality-adjusted life years (QALY; QALY’s were calculated using a general health-state patient-reported quality of life metric, the EuroQol Five-Dimensions questionnaire), ODI |
| Chan et al. (2021) | CO | • Clavien-Dindo grade IV complication (cardiac arrest, myocardial infarction, septic shock, pulmonary embolism, postoperative dialysis, need for reintubation, and prolonged ventilator requirements) |
|  |  | • Unplanned readmission, reoperation, and nonhome discharge |
| Chang et al. (2020) | CO | • Clavien-Dindo grade 2 (complications requiring pharmacological treatments with drugs other than analgesics, antiemetics, antipyretics, diuretics and electrolytes) or higher, general complication (delirium, cardiovascular (anginal, myocardial infarct, pulmonary edema), urinary (infection, retention), respiratory (pneumonia, pleural effusion), gastrointestinal (ileus), and fall (sacral fracture)), surgical (neurologic symptom (pain, weakness), infection, hematoma, and pneumoperitoneum) |
| Charest-Morin et al. (2018) | CO | • Intraoperative complications (dural tear, instrumentation failure, positioning-related complications) and postoperative complications (anemia, cardiac complications, wound infection, delirium, electrolyte abnormalities, pneumonia, neuropathic pain, urinary tract infection, urinary retention) |
|  |  | • LOS, nonhome discharge, mortality |
| Elsamadicy et al. (2021) | CO | • Medical complications (pneumonia, unplanned reintubation, requiring mechanical ventilation, pulmonary embolism, renal insufficiency, acute renal failure, urinary tract infection, coma, stroke, neurological deficit, cardiac arrest, myocardial infarction, deep vein thrombosis, clostridium difficile colitis, systemic sepsis, or septic shock) and surgical complications (superficial SSI, deep SSI, organ space SSI, wound dehiscence)  • LOS, reoperation, and readmission |
| Flexman et al. (2016) | CO | • Clavien-Dindo grade 2 or higher (cardiac arrest, myocardial infarction, sepsis or septic shock, stroke, pulmonary embolism, deep vein thrombosis requiring treatment, acute renal failure requiring treatment, acute renal failure requiring dialysis, urinary tract infection, pneumonia, wound dehiscence, deep incisional surgical site infection, organ-space surgical site infection, return to the operating room with 30 days, unplanned reintubation, and failure to wean from a ventilator >48 hours) |
|  |  | • LOS, nonhome discharge, mortality |
| Hannah et al. (2020) | CO | • Total complications, required intensive care unit stay, nonhome discharge, direct costs of hospitalization, readmission and emergency department visits |
| Jung et al. (2022) | CO | • Gastrointestinal complications (ileus, peritoneal injury, or bowel injury), urologic complications (retention, urinary tract infection, or incontinence), wound complications (dehiscence or infection), neurologic complications, graft failure (cage subsidence) |
|  |  | • adjacent segmentation degeneration, revision surgery, length of bed rest, and LOS |
|  | PRO | • VAS for back and leg pain, ODI, the proportions of patients who achieved substantial clinical benefit for VAS-back, VAS-leg (2.5-point change), and ODI (18.8-point change) |
| Kang et al. (2020) | CO | • Postoperative complications (urinary tract infection, pneumonia, surgical site infection, sepsis, deep vein thrombosis, pulmonary embolism, stroke, myocardial infarction, and cardio/pulmonary arrest) |
| Kim et al. (2020) | CO | • Postoperative complications (death, cardiac arrest, deep vein thrombosis, myocardial infarction, postoperative intubation, pulmonary embolism, stroke, sepsis, acute kidney injury, pneumonia, surgical infection, and urinary tract infection) |
| Kim et al. (2021) | CO | • Postoperative complications ($\geq$Clavien-Dindo grade Ⅱ)  • LOS |
| Leven et al. (2016) | CO | • Postoperative complications (pneumonia, sepsis, deep vein thrombosis, pulmonary embolism, wound complication, deep infection, central nervous system complication, sepsis/septic shock, cardiac arrest, acute renal failure, urinary tract infection, blood transufion, and reoperation) |
|  |  | • Mortality |
| Li et al. (2021) | CO | • Major postoperative complication by Glassman et al. (cardiac complications (myocardial infarction, congestive heart failure, atrial fibrillation, and malignant arrhythmia), pneumonia, delirium, stroke, neurological deficit, deep wound infection, acute renal dysfunction (increase in serum creatinine $\geq$26.5$\mu$mol/L or $>$1.5 times the baseline value within 48 hours), gastrointestinal adverse events (digestive tract hemorrhage and alimentary tract perforation), and deep vein thrombosis, or pulmonary embolism) |
|  |  | • Radiographic imaging |
|  | PRO | • ODI, SRS-22, JOA, VAS for back pain |
| Miller et al. (2017) | CO | • Major complications by Glassmane et al. (potentially life-threatening, required reoperation, or caused permanent injury; intraoperative vascular, visceral, or neurologic injury, deep wound infection, pulmonary embolism, junctional failure, and other similar complications) |
|  |  | • deep wound infection rate, wound dehiscence incidence, PJK incidence, pseudarthrosis, reoperation incidence |
|  |  | • LOS |
| Miller et al. (2018, Jan) | CO | • Major complications (life threatening or that could adversely affect the treatment outcome; intraoperative vascular, visceral, or neurologic injury, deep wound infection, pulmonary embolism, junctional failure), surgical complications (most intraoperative complications and immediate postoperative complications related to surgical technique/error), medical complications (unrelated to surgical technique, including stroke, deep venous thrombosis, pulmonary embolism, pneumonia, and urinary tract infection) |
|  |  | • ICU admission, LOS, nonhome discharge |
| Miller et al. (2018, Sep) | CO | • Major complications Glassman et al. and McDonnel et al. (substantially changed the expected path to recovery and were potentially life-threatening, required reoperation, or caused permanent injury; intraoperative vascular, visceral, or neurologic injury, deep wound infection, pulmonary embolism, junctional failure, and other similar complications) |
|  |  | • Proximal junctional kyphosis, deep wound infection, surgical complications (intraoperative and immediate postoperative complications), and medical complications (stroke, deep venous thrombosis, pulmonary embolus, pneumonia, and urinary tract infection) |
| Miller et al. (2018, Oct) | CO | • Major complications by Glassman et al. and McDonnel et al. (life threatening or that could adversely affect the treatment outcome; intraoperative vascular, visceral, or neurologic injury, deep wound infection, pulmonary embolism, junctional failure) |
|  |  | • LOS |
| Passias et al. (2019) | CO | • Any infection (Deep incisional SSI, superficial SSI, urinary tract infection, other infection), cardiopulmonary (pneumonia, respiratory failure, tachycardia, cardiac arrest, other cardiac event), neurologic (C5 motor deficit, mental status change, nerve root motor deficit, nerve sensory deficit, radiculopathy), gastrointestinal (ileus, perforated ulcer), vascular, instrumentation failure |
|  |  | • LOS, Nonhome discharge, reoperation, mortality |
|  | PRO | • NDI, NRS back and neck pain, EQ-5D |
| Passias et al. (2022) | CO | • All complications (infection, wound, implant-related, radiographic, neurological, gastrointestinal) |
|  | PRO | • ODI, SRS-22, EQ5D, pain catastrophizing scale |
| Phan et al. (2017) | CO | • Any complications (pulmonary complication (pneumonia, intubation, or ventilator requirement), renal complications (progressive renal insufficiency or acute renal failure), central nervous system (stroke or coma), cardiac complication (cardiac arrest or myocardial infarction), venous thromboembolism, urinary tract infections, sepsis, wound infection (superficial wound infection, deep incisional surgical site infection, organ space surgical site infection, or wound dehiscence), graft failure, or blood transfusion) |
|  |  | • Blood transfusion, LOS>5 days, reoperation |
| Pierce et al. (2019) | CO | • Complications (any complication, major, minor, intraoperative, excessive bleeding, cardiopulmonary, gastrointestinal, infection, instrumentation, neurologic, radiographic imaging, wound, dural tear, and pseudoarthrosis) |
|  |  | • LOS |
|  | PRO | • NRS back and leg pain, ODI, SRS-22 |
| Pierce et al. (2021, May) | CO | • DJK occurrence, major complication, reoperation |
|  |  | • Radiographic imaging |
|  | PRO | • NDI, NRS for neck pain, EQ5D, mJOA |
| Pierce et al. (2021, July) | CO | • Postoperative complication (septic shock, sepsis, deep venous thorombosis/thrombophlebitis, myocardial infarction, cardiac arrest, stroke/cerebrovascular accident complication, urinary track infection, acute renal failure, on a ventilator for >48 hours, pulmonary embolism, unplanned intubation, pneumonia, wound disruption, organ site, surgical site infection, deep incisional surgical site infection, or superficial surgical site infection)  • Unplanned readmission, mortality |
| Pierce et al. (2021, August) | CO | • Hospital acquired condition (Deep incisional SSI, wound disruption, pneumonia, unplanned intubation, pulmonary embolism, ventilator $>$48 hours, progressive renal insufficiency, acute renal failure, stroke/ CVA with neurological deficit, cardiac arrest requiring CPR, myocardial infarction, sepsis)  • LOS |
| Puilido et al. (2022) | CO | • Revision surgery rate, Clavien-Dindo Grade Ⅳ complications, blood transfusion, internal complications, thromboembolisms, apoplexy, delirium, syncope and collapse, surgical site infection rates |
| Reid et al. (2018) | PRO | • ODI, SF-36 PCS, NRS back and leg |
|  |  | • Substantial clinical benefit by Glassman et al. (8.8-point change in ODI score, 6.2-point change in SF-36 PCS score, 2.5-point change in back pain score, and 2.5-point change in leg pain score) |
| Rothrock et al. (2019) | CO | • Frail status (phenotype) |
|  | PRO | • Postoperative Quality of Recovery Scale ( for cognitive recovery and ADL) |
|  |  | • Alzheimer’s disease research center IADL |
| Shahrestani et al. (2021) | CO | • Postoperative complications queried within the primary discharge (posthemorrhagic anemia, infection, urinary tract infection) |
|  |  | • Complications at readmission (infection, acute kidney failure, thromboembolic, wound dehiscence, hardware failure, neurological injury) |
|  |  | • Mean all-payer cost, nonhome discharge, LOS |
|  |  | • Mortality, readmission rate |
| Shin et al. (2017) | CO | • Clavien-Dindo grade IV complication (life-threatening conditions involving single or multiorgan dysfunction requiring intermediate care of intensive care unit management; cardiac arrest, myocardial infarction, septic shock, pulmonary embolism, postoperative dialysis, need for reintubation, and prolonged ventilator requirements), hospital-acquired conditions (surgical site infection, urinary tract infection, and venous thromboembolism)  • Mortality |
| Sun et al. (2020) | CO | • Rate of any complications (Intra-operative complications (dural tear, instrumentation failure, and positioning related), postoperative complications (pneumonia, sepsis, deep vein thrombosis, pulmonary embolism, surgical site infections, central nervous system complication, sepsis/septic, cardiac arrest, acute renal failure, and urinary tract infections) |
|  |  | • LOS, nonhome discharge, reoperation, readmission |
|  |  | • Mortality |
|  |  | • Major complication (life threatening or that could adversely affect the treatment outcome), and minor complication (that did not compromise outcomes) |
|  | PRO | • ODI, SF-36 |
| Susano et al. (2020) | CO | • Delirium |
|  |  | • All other-cause complications (cardiopulmonary (myocardial infarction, congestive heart failure, cardiac arrest, new onset arrhythmia, pulmonary embolism, reintubation, and deep venous thrombosis), infectious (wound infections, pneumonia, sepsis, and urinary tract infection), renal (acute renal injury), or cerebrovascular (stroke and transient ischemic accident) |
|  |  | • LOS and nonhome discharge |
| Ton et al. (2022) | CO | • Postoperative complications (acute posthemorrhagic anemia, infection, urinary tract infection) |
|  |  | • Complications at readmission (infection, acute kidney failure, thromboembolic, wound dehiscence, hardware failure, neurological injury) |
|  |  | • Mean all-payer cost, nonhome discharge, LOS |
|  |  | • Mortality, readmission rate |
| Weaver et al. (2019) | CO | • Medical complications (pneumonia, unplanned intubation, pulmonary embolism, postoperative ventilator use, progressive renal insufficiency, acute renal failure, urinary tract infection, CVA/stroke, cardiac arrest, myocardial infarction, bleeding transfusion, DVT/thrombophlebitis, sepsis, septic shock) |
|  |  | • Mortality, nonhome discharge, readmissions |
| Wilson et al. (2020) | CO | • Major complication (pneumonia, deep vein thrombosis, pulmonary embolism, myocardial infarction, cardiac arrest, wound infection or dehiscence, and sepsis) |
|  |  | • LOS, mortality, nonhome discharge, unplanned readmission, unplanned reoperation |
| Yagi et al. (2018) | CO | • Total complication rate |
|  |  | • Radiographic imaging |
|  | PRO | • ODI, SF-36, SRS-22, and pain |
| Yagi et al. (2019) | CO | • Major complications (neurological, implant-related (proximal and distal junctional kyphosis, rod breakage, pseudoarthrosis, implant dislodgement, screw breakage, and others), surgical-site infection, other infection (urinary tract infection and others), excessive bleeding (>2000mL), delirium, cardiopulmonary (hemodynamic instability, myocardial infarction, deep venous thrombosis, pulmonary embolisms, thoracic atelectasis, congestive heart failure, and others), gastrointestinal (ileus, cholecystitis), and renal (acute renal failure)) |
|  |  | • SAEs (Clavien-Dindo grade $\geq$3, reoperation required, deterioration of motor function at discharge, or new motor deficit) |
| Zreik et al. (2021) | CO | • Minor (superficial incisional surgical site infection, pneumonia, urinary tract infection, and wound disruption) and major complications (myocardial infarction, renal insufficiency or failure, deep vein thrombosis, pulmonary embolism, deep incisional SSI, organ/space SSI, sepsis, and unplanned intubation), and any complications (combined minor and major complications) |
|  |  | • Extended LOS, nonhome discharge, unplanned readmission |

ADL, activities of daily living; EQ-5D, EuroQol-5D; HFRS, Hospital Frailty Risk Score; IADL, instrumental activities of daily living; ICU, intensive care unit; SF-36, 36-Item Short Form Survey; SRS-22, Scoliosis Research Society 22-question; JOA, Japanese orthopedic association scale; PQRS, Postoperative Quality of Recovery Scale ; ODI, Oswestry disability index; QALY, Quality-adjusted life years; SAEs, severe adverse events; LOS, length of hospital stay; NDI, Neck Disability Index; NRS, numerical rating scale; VAS, visual analogue scale.

**Supplementary Material Table 3. The incidence rates of complication in the robust and frail group**

| **Frail** | | | **Robust** | | |
| --- | --- | --- | --- | --- | --- |
|  | **Complications** | **Incidence rate** |  | **Complications** | **Incidence rate** |
| **1** | Implant-related complication | 21.5 % | **1** | Gastrointestinal complication | 5.6 % |
| **2** | Neurological complication | 13.6 % | **2** | Urinary tract infection | 4.6 % |
| **3** | Urinary tract infection | 9.3 % | **3** | Implant-related complication | 1.5 % |
| **4** | Gastrointestinal complication | 5.6 % | **4** | Neurological complication | 1.4 % |
| **5** | Stroke/CVA | 2.1 % | **5** | Superficial SSI | 0.6 % |
| **6** | Pneumonia | 1.3 % | **6** | Pneumonia | 0.5 % |
| **7** | Superficial SSI | 1.1 % | **7** | Deep vein thrombosis | 0.4 % |
| **8** | Sepsis | 0.8 % | **8** | Sepsis | 0.4 % |
| **9** | Myocardial infarction | 0.7 % | **9** | Deep wound infection | 0.3 % |
| **10** | Deep wound infection | 0.7 % | **10** | Pulmonary embolism | 0.3 % |
| **11** | Deep vein thrombosis | 0.6 % | **11** | Mortality | 0.2 % |
| **12** | Pulmonary embolism | 0.5 % | **12** | Myocardial infarction | 0.1 % |
| **13** | Mortality | 0.4 % | **13** | Stroke/CVA | 0.1 % |
| **14** | Cardiac arrest | 0.3 % | **14** | Cardiac arrest | 0.1 % |
| **15** | Acute renal failure | 0.3 % | **15** | Acute renal failure | 0.1 % |

CVA= cerebrovascular accident; SSI= surgical site infection.

**Supplementary Material Figure 2.** Forest plots for each clinical outcome

| *2.1. Mortality* |
| --- |
|  |
| Event= mortality |
|  |
| *2.2. Major complication* |
|  |
| Event= major complication  “Shin et al. (2017) a” and “Shin et al. (2017) b” are the same article. |
|  |
| *2.3. Any complication* |
|  |
| Event= any complication |
|  |
| *2.4. General complication* |
|  |
| Event= general complication |
| *2.5. Acute renal failure* |
|  |
| Event= acute renal failure |
|  |
| *2.6. Cardiac arrest* |
|  |
| Event= cardiac arrest |
|  |
| *2.7. Deep vein thrombosis* |
|  |
| Event= deep vein thrombosis |
| *2.8. Gastrointestinal complication* |
|  |
| Event= gastrointestinal complication |
|  |
| *2.9. Myocardial infarction* |
|  |
| Event= myocardial infarction |
|  |
| *2.10. Pneumonia* |
|  |
| Event= pneumonia |
|  |
| *2.11. Pulmonary embolism* |
|  |
| Event= pulmonary embolism |
| *2.12. Sepsis* |
|  |
| Event= sepsis |
|  |
| *2.13. Stroke/* cerebrovascular accident |
|  |
| Event= stroke/cerebrovascular accident |
|  |
| *2.14. Urinary tract infection* |
|  |
| Event= urinary tract infection |
|  |
| *2.15. Surgical complication* |
|  |
| Event= surgical complication |
| *2.16. Deep wound infection* |
|  |
| Event= deep wound infection |
|  |
| *2.17. Implant-related complications* |
|  |
| Event= implant-related complications |
|  |
| *2.18. Neurologic complications* |
|  |
| Event= neurologic complications |
|  |
| *2.19. Superficial surgical site infection* |
|  |
| Event= superficial surgical site infection |
|  |
| *2.20. Nonhome discharge* |
|  |
| Event= nonhome discharge |
| *2.21. Reintubation* |
|  |
| Event= reintubation |
|  |
| *2.22. Reoperation* |
|  |
| Event= reoperation |
| *2.23. Length of hospital stay* |
|  |
|  |

**Supplementary Material Figure 3.** Forest plots for the patient-reported outcome of the meta-analyses

**Supplementary Material Figure 4.** Forest plot after sensitivity analyses for any complication

**Supplementary Material Figure 5.** Forest plot for any complication after removing outlier studies
